# Neural mechanisms of peristalsis in the isolated rabbit distal colon: a neuromechanical loop hypothesis

**DOI:** 10.3389/fnins.2014.00075

**Published:** 2014-04-16

**Authors:** Phil G. Dinning, Lukasz Wiklendt, Taher Omari, John W. Arkwright, Nick J. Spencer, Simon J. H. Brookes, Marcello Costa

**Affiliations:** ^1^Department of Gastroenterology and Surgery, Flinders Medical Centre, Flinders UniversityBedford Park, SA, Australia; ^2^Department of Human Physiology, School of Medicine, Flinders UniversityBedford Park, SA, Australia; ^3^Gastroenterology Unit, Child, Youth and Women's Health ServiceAdelaide, SA, Australia; ^4^Material Science and Engineering, CSIROSydney, NSW, Australia

**Keywords:** colon motility, peristalsis, neural pathways, active contraction, active relaxation

## Abstract

Propulsive contractions of circular muscle are largely responsible for the movements of content along the digestive tract. Mechanical and electrophysiological recordings of isolated colonic circular muscle have demonstrated that localized distension activates ascending and descending interneuronal pathways, evoking contraction orally and relaxation anally. These polarized enteric reflex pathways can theoretically be sequentially activated by the mechanical stimulation of the advancing contents. Here, we test the hypothesis that initiation and propagation of peristaltic contractions involves a neuromechanical loop; that is an initial gut distension activates local and oral reflex contraction and anal reflex relaxation, the subsequent movement of content then acts as new mechanical stimulus triggering sequentially reflex contractions/relaxations at each point of the gut resulting in a propulsive peristaltic contraction. In fluid filled isolated rabbit distal colon, we combined spatiotemporal mapping of gut diameter and intraluminal pressure with a new analytical method, allowing us to identify when and where active (neurally-driven) contraction or relaxation occurs. Our data indicate that gut dilation is associated with propagating peristaltic contractions, and that the associated level of dilation is greater than that preceding non-propagating contractions (2.7 ± 1.4 mm vs. 1.6 ± 1.2 mm; *P* < 0.0001). These propagating contractions lead to the formation of boluses that are propelled by oral active neurally driven contractions. The propelled boluses also activate neurally driven anal relaxations, in a diameter dependent manner. These data support the hypothesis that neural peristalsis is the consequence of the activation of a functional loop involving mechanical dilation which activates polarized enteric circuits. These produce propulsion of the bolus which activates further anally, polarized enteric circuits by distension, thus closing the neuromechanical loop.

## Introduction

The most common form of intestinal propulsion, often described as peristalsis, is due to the aboral propagation of rings of circular muscle contractions (peristaltic contractions), and as it involves polarized enteric neural circuits it can be called “neural peristalsis” (Furness and Costa, 1987; Brookes, 2001a; Brookes and Costa, 2002a, 2006; Bornstein et al., 2004; Furness et al., 2004). An early observation (Mall, 1896) showed that a stimulus locally applied to the gut wall causes contraction above the stimulus and relaxation below. The terms ascending excitatory and descending inhibitory reflexes have been widely used to describe these polarized responses. In Bayliss and Starling (1899) postulated the existence of an anatomical basis of these polarized enteric reflex pathways where they concluded that “the system is composed of long paths which conduct inhibitory impulses downwards, and short paths which carry augmentor impulses from one cell-station to another in an upward direction.” Nearly 100 years later such histological evidence has been provided. Indeed both the final enteric excitatory and inhibitory motor neurons to the circular muscle have unique local/oral and aboral polarities respectively and specific classes of ascending and descending interneurons are involved in these reflex pathways (Brookes and Costa, 2002b).

The relationship between simple polarized reflexes and propagating peristaltic contractions has been the subject of speculation. (Mall, 1896) Bayliss and Starling (Bayliss and Starling, 1899) proposed that “for the onward progress of the bolus two factors are equally necessary, namely a condition of excitation and increased contraction above the bolus, and a condition of inhibition and relaxation of the intestine below.” They termed this the “law of the intestine” and stated that “the peristaltic contraction is a true *coordinated reflex*, excited by the distension of the gut at some point, or perhaps, when once established, by the advancing line of constriction itself.” This has intuitive appeal, however, experimental findings have demonstrated otherwise. The term “peristaltic reflex” (or “myenteric reflex” Cannon, 1911), has been widely used to describe the entire process of “peristalsis” i.e., propagating contractions that results in propulsion of contents. However, peristalsis seems to be a more complex, coordinated motor behavior, which is triggered at a discrete threshold and which then involves sequential activation of enteric circuits, rather than comprising a single, simple reflex (Tonini et al., 1996). Mechanical and electrophysiological recordings of circular muscle, in many isolated preparations of colon (Auer and Krueger, 1947; Hukuhara and Miyake, 1959; Hukuhara and Neya, 1968; Costa and Furness, 1976; Jule, 1980; Grider and Makhlouf, 1986; Messori et al., 1995; Bian et al., 2003; Spencer et al., 2003, 2011) [but also in the intact human colon (Bjornsson et al., 1998; Spencer et al., 2012)], clearly confirm that localized distension initiates polarized ascending excitatory and descending inhibitory reflex responses (standing enteric reflexes). However, despite abundant histological and functional evidence for the existence of polarized enteric reflexes, understanding how they contribute to propulsive peristaltic contractions is still largely lacking.

## The neuromechanichal loop hypothesis

As neural peristalsis is a behavior involving enteric neural circuits that are dependent on ongoing mechanical stimulation of the advancing contents, it could be considered as a self-sustaining propulsive behavior. In this study we call this description of events the “***neuromechanical loop hypothesis***” of neural peristalsis. This hypothesis places the enteric reflex pathways as the basis of peristalsis, and places them in a dynamic process, thus avoiding the historical misconception of peristalsis as a simple “reflex.” This hypothesis requires activation of excitatory neurons by mechanical distension to initiate the **peristaltic contractions**. Furthermore, once the peristaltic contractions starts, the mechanical distension, created by propulsion of content, immediately anal to the contracted region, should act as a new mechanical stimulus, activating the polarized enteric reflex pathways in sequence but displaced aborally, resulting in the peristaltic behavior with associated propulsion of contents.

Ideally, this hypothesis could be tested by recording from excitatory and inhibitory enteric motor neurons during periods of propagating contractions associated with movement of content. However, the very movements of the gut wall pose serious methodological constraints. One study that did succeeded in recording, from a fixed point, electrical activity from of smooth muscle during peristalsis in a tubular preparation had to remove the serosa and longitudinal muscle and because recording was limited to one location the site of origin of the motor neuron input could not be determined (Yokoyama and North, 1983).

We have recently developed a method, combining spatiotemporal maps of gut diameter and intraluminal pressure that identifies, in tubular preparations, where the smooth muscle is being actively excited or inhibited (Costa et al., 2013b) and where it is likely to be responding passively to movement of content from elsewhere. Using that technique, in this paper we provide evidence for the neuromechanical hypothesis of neural peristalsis.

## Methodology and results

### Experimental setup

Six New Zealand albino rabbits of both sexes weighing 2–4 kg were euthanized humanely by intravenous injection of sodium pentobarbitone (0.5 ml/kg) in accordance with approval by the Animal Welfare Committee of Flinders University. A ventral midline incision was made to expose the peritoneal cavity. As in our previous studies (Dinning et al., 2012; Costa et al., 2013b) we used segments of rabbit distal colon, 15–27 cm long. These were placed into beakers containing oxygenated Krebs solution (in mM: NaCl, 118; KCl, 4.7; NaH_2_PO_4_, 1.0; NaHCO_3_, 25; MgCl_2_, 1.2; D- Glucose, 11; CaCl_2_, 2.5) and bubbled with 95% O_2_/5% CO_2_. The fecal pellets were gently flushed out of the colonic segments with Krebs solution.

The experimental setup used in this preparation has been described in previous publications (see Figure 1 in Costa et al., 2013b). Briefly each colonic segment was placed into an organ with the oral and anal ends attached to a T-shaped plastic connector. A fiber-optic manometry catheter (Arkwright et al., 2009) was then inserted into the colonic segment through the oral T connector. The catheter tip was pushed through the entire segment until it was located in the anal end T connector. Parafilm wax (Benis Flexible packaging, Neenah, USA) was wrapped around the catheter and T piece at either end to prevent the isolate the luminal contents from the organ bath. The remaining arm of the oral T piece was connected to an infusion pump through which warmed (35°C) Krebs solution could be infused. All studies were done in a closed preparation (i.e., fluid ejection from the anal end was prevented). Both the oral and anal T pieces were fixed to the base of the organ bath to prevent net shortening of the preparation.

### Fiber-optic manometry recording

The manometry catheter incorporated 60 sensors, spaced at 1 cm intervals, and had an outside diameter of 3 mm (Arkwright et al., 2009). The catheter was attached to a spectral interrogator unit (FOS&S FBG-scan 804. FOS&S, Geel, Belgium) and pressures were recorded in real time using a custom written LabVIEW program (National Instruments, TX, US). These recordings of intraluminal pressure were used to create spatiotemporal pressure maps (PMaps; see Box 1).

Box 1 **Method to identify excitatory and inhibitory enteric motor neurons input to smooth muscle**

**Constructions of diameter maps (DMaps) and pressure maps (PMaps)**

**Figure 1:**
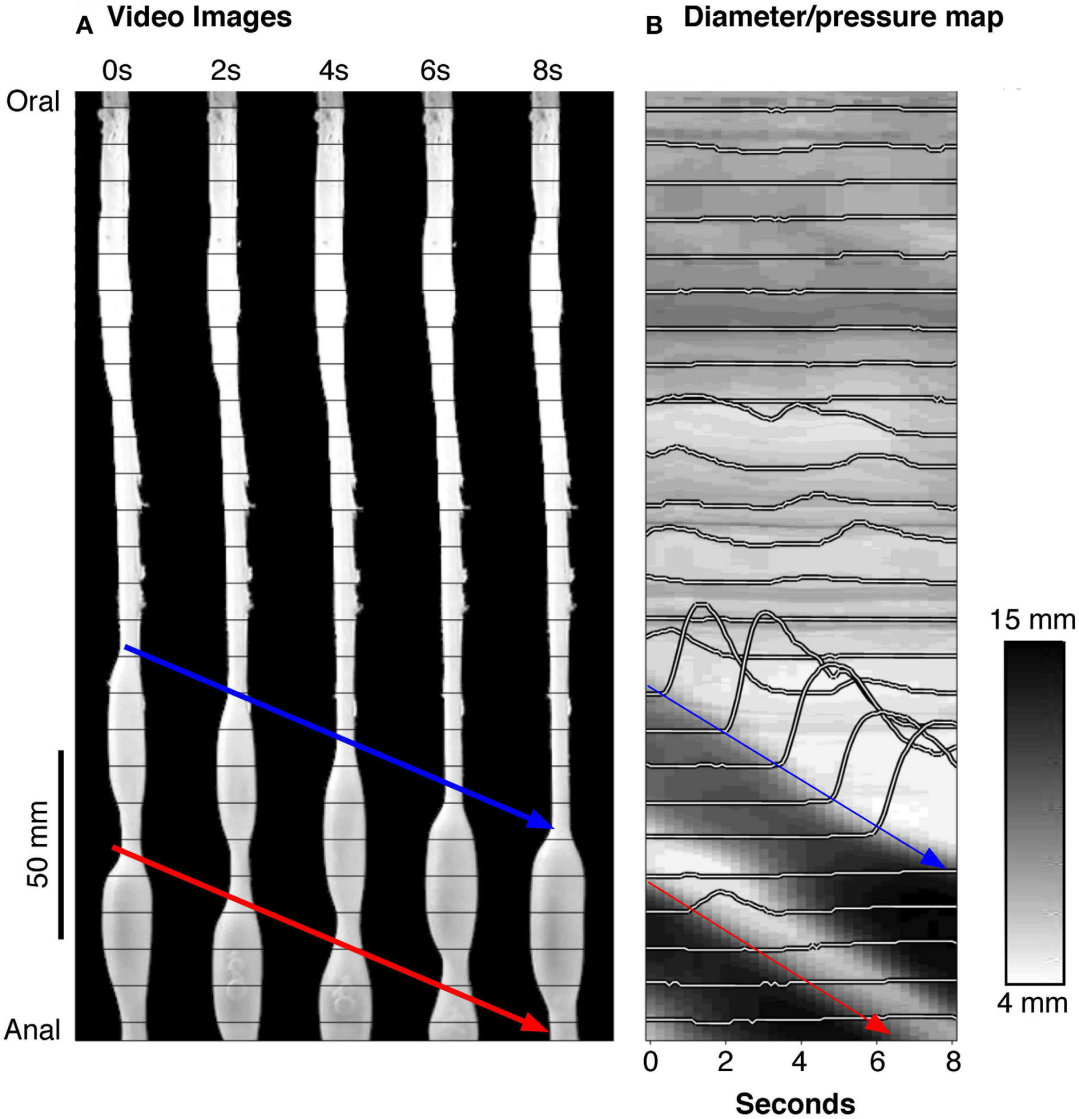
**(A)** A series of silhouettes of the rabbit colon taken at 2 s intervals during the movement of a bolus. The thin horizontal lines indicate the location of the fiber-optic pressure sensors (at 10 mm intervals). **(B)** A spatiotemporal diameter map (DMap) created from the same 8 s period with the multichannel intraluminal pressure traces (recorded by the fiber-optic catheter) superimposed. The blue and red arrows represent individual bolus movement in both **(A,B)**. On the DMap **(B)**, the movement of the bolus is represented by the oblique dark bands (representing distension). At the blue arrow in **(B)**, it is clear that the onset of contraction is associated with large pressure waves (recorded by the catheter sensors).

The method developed to identify when and where gut smooth muscle is excited or inhibited relies on combining spatiotemporal maps of gut diameter with maps of intraluminal pressure (Costa et al., 2013b). Videos of rabbit colon were converted to spatio-temporal maps of changes in diameter (“Dmaps”) with software written in Matlab, adapted from the methods developed in our laboratory (Hennig et al., 1999). Briefly, videos were thresholded, converted to black and white; the diameter at each point was calculated for each frame and converted into either a gray or color scale, to create a spatio-temporal map of diameter changes (Figure 1). The pressure recorded by the fiber-optic catheter were then aligned in both space and time (Figure 1) with the DMaps, down-sampled to 4 Hz (to match the Dmap) and interpolation between sensors used to create an equivalent pressure map (Pmap).

**Use of diameter/pressure maps (DPmaps)**

**Figure 2:**
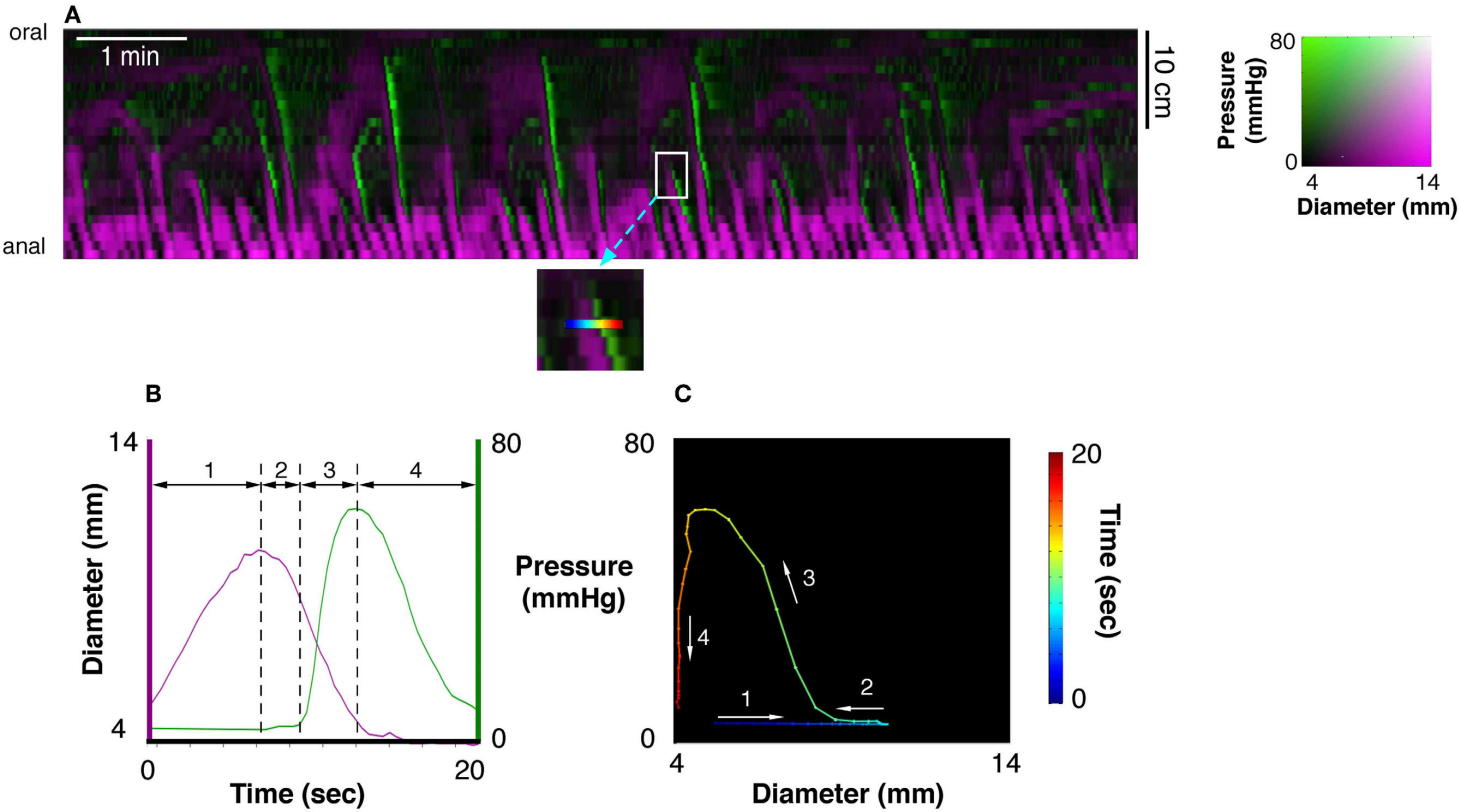
**(A)** A 10 min spatiotemporal diameter/pressure map (DPMap) recorded over a 25 cm long piece of rabbit colon during a basal period in one rabbit. Intraluminal pressure is shown as black (low pressure) to green (high pressure) and the changes in diameter are similarly shown in shades of magenta. The white box in **(A)**, is shown expanded (inset below the DPMap) and a rainbow colored line in it marks 20 s at one site used for subsequent analysis. The line plots, of pressure (green) and diameter (magenta) from the inset are plotted against time in **(B)**, and against each other in the orbit plot **(C)**. The rainbow-colored line in the inset (from blue via green, yellow to red) represents the 20 s period, which form 4 distinct regions in the pressure/diameter plot. First (where the line is blue-cyan), diameter increases [magenta line in **(B)**] with no change in pressure [green line in **(B)**]. In the 2nd stage (cyan to green) diameter begins to decrease with no change in pressure. In the 3rd stage (green to yellow) pressure begins to increase with a corresponding decrease in diameter. In the 4th stage (orange to deep red) the diameter decreases, with little change in diameter. The orbit plot represents each of the 4 stages and thus defines the succeeding states of the muscle at this one point in the preparation over a 20 s period.

PMaps with DMaps were then pseudo-colored and combined (Costa et al., 2013b). These aligned diameter/pressure maps (DPMaps) allowed pressure and diameter (in different colors) to be visualized on the same map (Figure 2A). A more detailed description of the creation of the DPMaps can be found in our previous paper (Costa et al., 2013b). For each motility event (measured at a single point along the preparation, over a few seconds see Figure 2B) it was possible to extract and plot pressure values against diameter values. These could then be expressed as an orbital plot (Figure 2C). Each part of the orbit plot represented a changing mechanical state of the muscle (see arrows Figure 2C). We assumed that the colon was circular in cross-section. Changes of the mechanical states, deduced from orbit plots were calculated using a Hidden Markov Model (Costa et al., 2013b; Wiklendt et al., 2013). Twelve mechanical states could be distinguished from orbit plots, as described previously (Costa et al., 2013b). These mechanical states were divided into (A) active contractions, (B) active relaxations, and (C) passive states.

**Figure 3:**
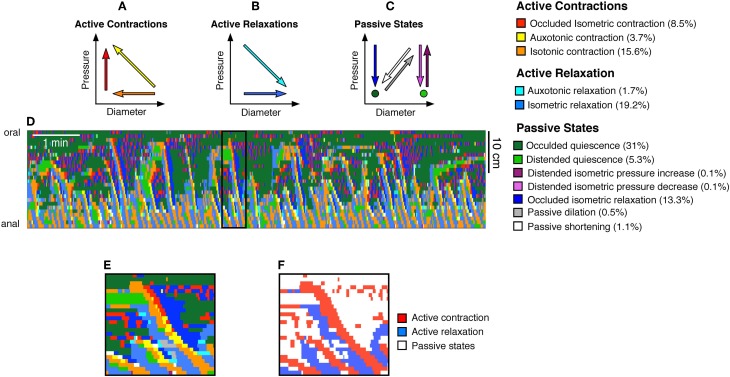
**From the orbital plots, 12 mechanical states were identified as described before (Costa et al., 2013b)**. These mechanical states were divided into **(A)** active contractions, **(B)** active relaxations, and **(C)** passive states. The color coded arrows shown in **(A–C)** indicate the trajectories of pressure and diameter which define each state. For example, the red arrow in **(A)** indicates an increase in pressure with no change in diameter, and this mechanical state represents an occluded isometric contraction. Taking the same DPMap shown in Figure 2A we can then determine the location and timing of each of these states and superimpose them upon the map **(D)**. The numbers in brackets at the end of the mechanical state descriptors indicate the percentage time occupied by each state in this spatiotemporal map. This map **(D)** is complex to interpret but can be readily simplified by identifying just two types of states: active contraction **(**3 states shown in **A)** and active relaxation **(**2 states in **B)**. This has been carried out for the black outlined area in **(D)**, shown on a longer timebase in **(E,F)** in which active contraction is red, active relaxation is blue and other passive states are white.

Active Contractions (see Figure 3A) comprised:

*Occluded isometric contractions*: where the gut is at its minimum diameter and a squeeze increases pressure with no further reduction in diameter (red arrow Figure 3A).
*Auxotonic contractions*: when the gut starts to contract (decreasing diameter) resulting associated with increasing pressure (yellow arrow Figure 3A).
*Isotonic contractions*: were identified by a contraction (decrease in diameter) with no measurable change in local pressure (orange arrow, Figure 3A).

Active relaxations (Figure 3B) were identified as:

*Auxotonic relaxations*: when the gut begins to relax (increasing diameter) and the pressure simultaneously drops (cyan arrow Figure 3B).
*Isotonic relaxations*: when the gut begins to relax (increasing diameter) and the pressure does not change (blue arrow Figure 3B).

Passive states (Figure 3C) included:

*Occluded isometric relaxations*: when the gut was at its minimum diameter, a squeeze ended, causing a decrease in pressure, but no change in diameter (dark blue arrow Figure 3C).
*Distended isometric pressure increases*: these occurred when a region of gut was near its maximum diameter, but active contraction further orally drove fluid into the segment, while outflow was restricted downstream. Under these circumstances pressure increases with minimal detectable change in diameter (dark purple arrow Figure 3C).
*Distended isometric pressure decreases*: this occurred when the gut was near its maximum diameter and pressure decreases due to a loss of contraction further orally, with negligible changes in diameter. This typically follows the previous scenario—when the active contraction further orally ceases. (light purple arrow Figure 3C).
*Passive shortenings:* these occur when both the diameter and pressure decrease due to removal of downstream resistance (allowing the gut to passively return to resting diameter according to its natural elasticity) (white arrow Figure 3C).
*Passive dilations*: these are associated with simultaneous increases in both diameter and pressure, usually due to propulsion of material by an upstream active contraction, while there is resistance to outflow from the region of interest (gray arrow Figure 3C).
*Occluded quiescence*: This is when the gut is close to its minimum diameter and changes in pressure are not measurable (dark green circle Figure 3C).
*Distended quiescence:* occurs when the gut is near its maximal diameter and is not showing any changes in intraluminal pressure at the site of the orbit plot (light green circle Figure 3C).

These mechanical states can be calculated for every region in a DPmap and used to create a new muscle state map (Figure 3D).

**Defining periods of quiescence**

**Figure 4:**
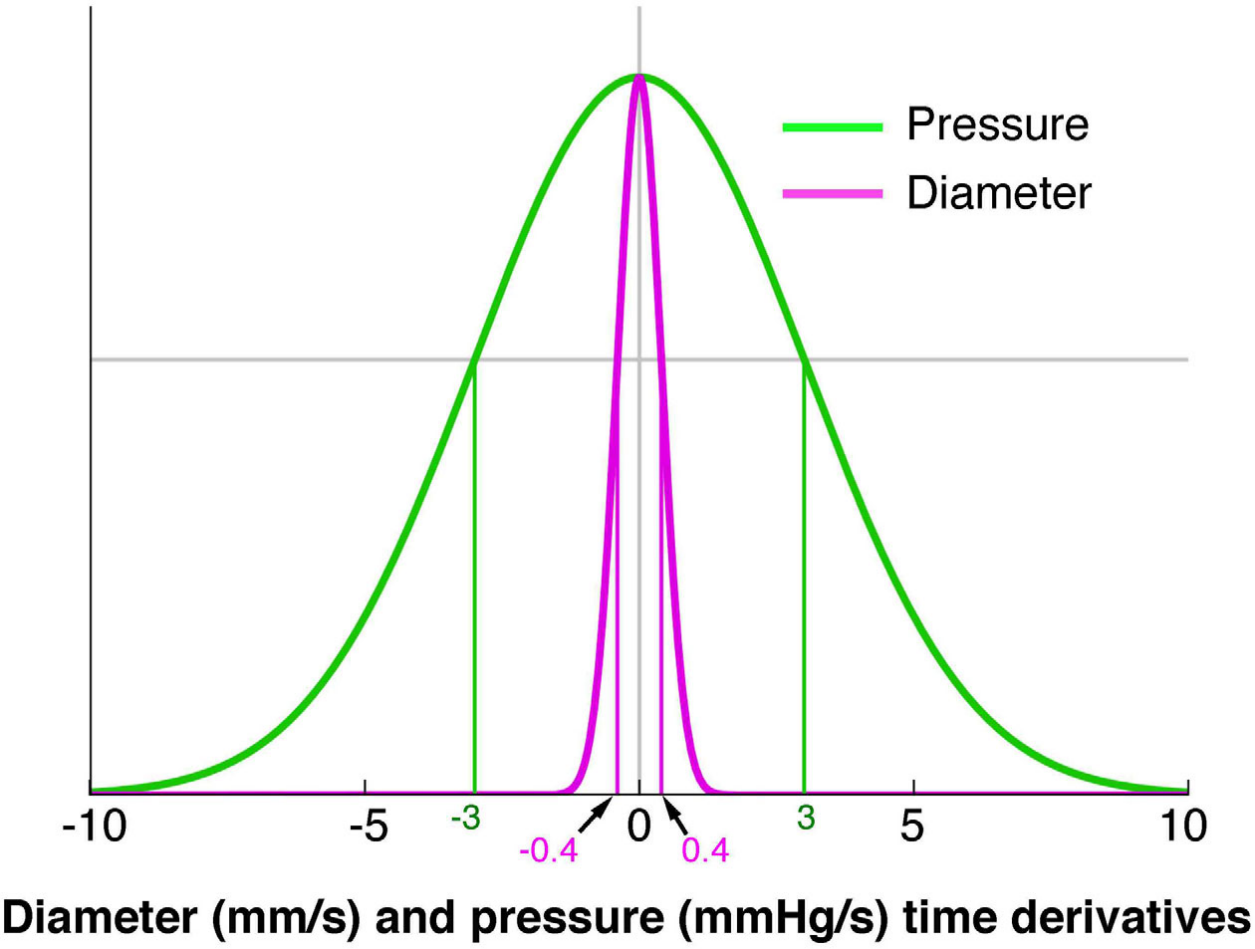
A model of the probability of a mechanical state being regarded as quiescent. The green line represents pressure and the magenta represents diameter. The x axis indicates the diameter (mm/s) or pressure (mmHg/s) time derivatives. The y axis represents conditional probability of quiescence at a given diameter or pressure time derivative. A value of 0 for either pressure or diameter indicates a strong probability of a quiescent state. A Hidden Markov Model was used to determine when a state moved from quiescence to activity (Wiklendt et al., 2013). Normalizing the pressure and diameter data was achieved by dividing each value by a constant defining the width of the quiescence model conditional probability curve (shown at vertical green and magenta lines). The constant for pressure was 3 mmHg/s and the constant for diameter was 0.4 mm/s.

A core component of our model is the ability to infer the mechanical state of the muscle from DPmaps. The difference between an auxotonic contraction (yellow arrow Figure 3A) and isotonic contraction (orange arrow Figure 3A) is that the increase in pressure for the orange arrow falls below a cut-off (Figure 4). In contrast, the yellow arrow defines an orbit trajectory in which changes in both diameter and pressure exceed threshold values. The probability for an observation to fall within any orbital trajectory is based on a Gaussian function (Figure 4) with details presented elsewhere (Wiklendt et al., 2013).

### Video recording of diameter changes

A digital video camera (Canon Legria HF S20. Ota, Tokyo, Japan), positioned above the preparation was used to record movies of colonic wall motion in clips of 10 min duration. These were then re-sampled down to 4 frames per second in Quicktime (Apple Inc. Cupertino, CA, USA). These video recordings were used to create spatiotemporal maps of changes in diameter (DMaps; see Box 1).

### Experiment protocol

With the fiber-optic catheter in the lumen, the gut was slowly distended (8 ml/min) by warmed (35°C) Krebs solution infused via the oral cannula. The maximal diameter it reached was calculated to correspond to its normal diameter when full of feces. Recordings of video and manometry started within 20 min of the onset of distension. As reported previously (Dinning et al., 2012) distension of closed preparations reliably elicited peristaltic (anally-propagating) contractions.

A timing light emitting diode, driven by recorded current pulses, was placed within the video field to allow synchronization between pressure traces and diameter recordings. A ruler placed adjacent to the preparation was used to identify the locations of manometry sensors. After initial recordings, tetrodotoxin (Tetrodotoxin to a final concentration of 0.6 μM) was added to block all neural activity.

### Construction of spatio-temporal maps of excitatory and inhibitory neural activity

Box 1 provides a summary of the steps taken in creating individual DMaps and PMaps and then combining them to create spatiotemporal maps of pressure and diameter (DPMaps). From these DPMaps orbital plots of pressure vs. diameter were calculated for all points as described previously (Costa et al., 2013b; Box 1). Using hidden Markov models, 12 different states of the muscle could be distinguished; 3 of which were combined and described as active excitation of muscle and 2 were combined to identify active relaxation (see Box 1). We assume that active excitation corresponds to excitatory input from enteric excitatory motor neurons and that active inhibition reflects input from enteric inhibitory motor neurons.

Our technique focuses upon neurogenic activity and while myogenic propagating contractions, generated by the pacemaker cells system (myogenic peristalsis) can also occur, in this work we have not encountered examples of it. As a result in this paper all spontaneous myogenic movements have been removed from the analysis. The techniques used to achieve this have been detailed in Box 2. With myogenic activity removed as well as any passive states (see Box 1) the resultant spatiotemporal map consisted of only regions of active contractions and relaxations. Six such spatiotemporal maps of rabbit distal colon are shown in Figure 7. From these maps all active propagating and non-propagating contractions were identified. An active propagating contraction was defined as a red region on the spatiotemporal maps of active contractions (Figure 7) that extended ≥20 mm (based on 3 pressure sensors spaced at 10 mm interval). All other active contractions were deemed non-propagating.

Box 2 **Constructing spatiotemporal maps of neurogenic active states of smooth muscle**

**Figure 5:**
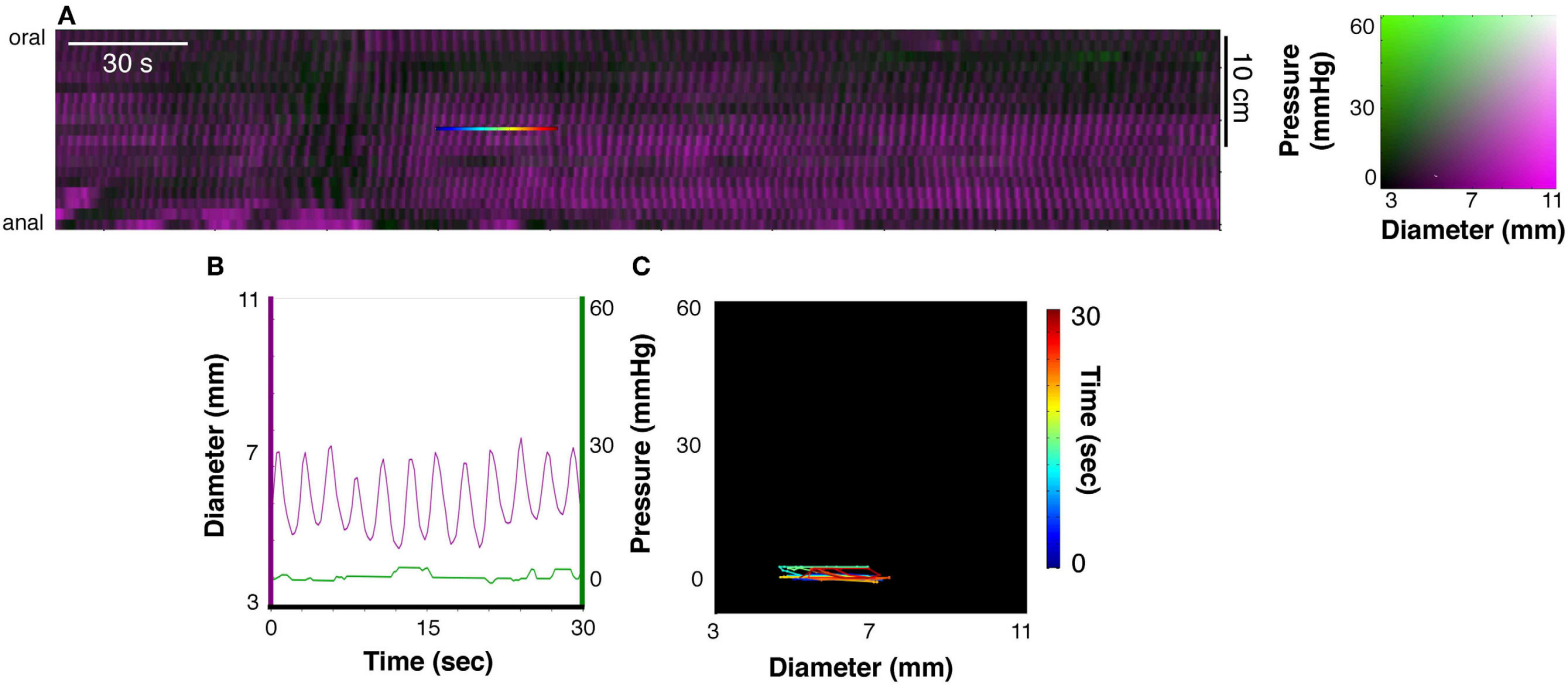
**(A)** Shows a DPMap of the rabbit colon after the addition of TTX (0.6 μM). All neurogenic activity is abolished, leaving only myogenic activity. Note that auto-scaling of the colors representing diameter (magenta) and pressure (green) exaggerate the amplitude of activity. Similar to Figure 2, the line plots, pressure and diameter, in **(B)** and the orbit plot in **(C)** have been taken from the rainbow-colored line in **(A)**. In this example the myogenic activity consists of low-amplitude, rhythmic changes in gut diameter [magenta line in **(B)**, with minimal effect on intraluminal pressure see **B**]. When plotted against each other, these give rise to flat orbit plots with short excursions in **(C)**.

As described in Box 1, DPmaps showed active contractions, active relaxations and passive states (Figure 3F). We were interested in neurogenic active states: this necessitated removal of myogenic changes in muscle state. Myogenic activity was identified by blocking all neural activity with tetrodotoxin (Dinning et al., 2012; Costa et al., 2013b). The resultant spatiotemporal maps of myogenic activity (Figure 5A) consisted of regular small changes in diameter (Figure 5B), presumably entrained by non-neuronal pacemaker cells; the Interstitial Cells of Cajal. Myogenic changes in gut diameter were minimal, and thus created orbit plots with short lengths (Figure 5C).

**Figure 6:**
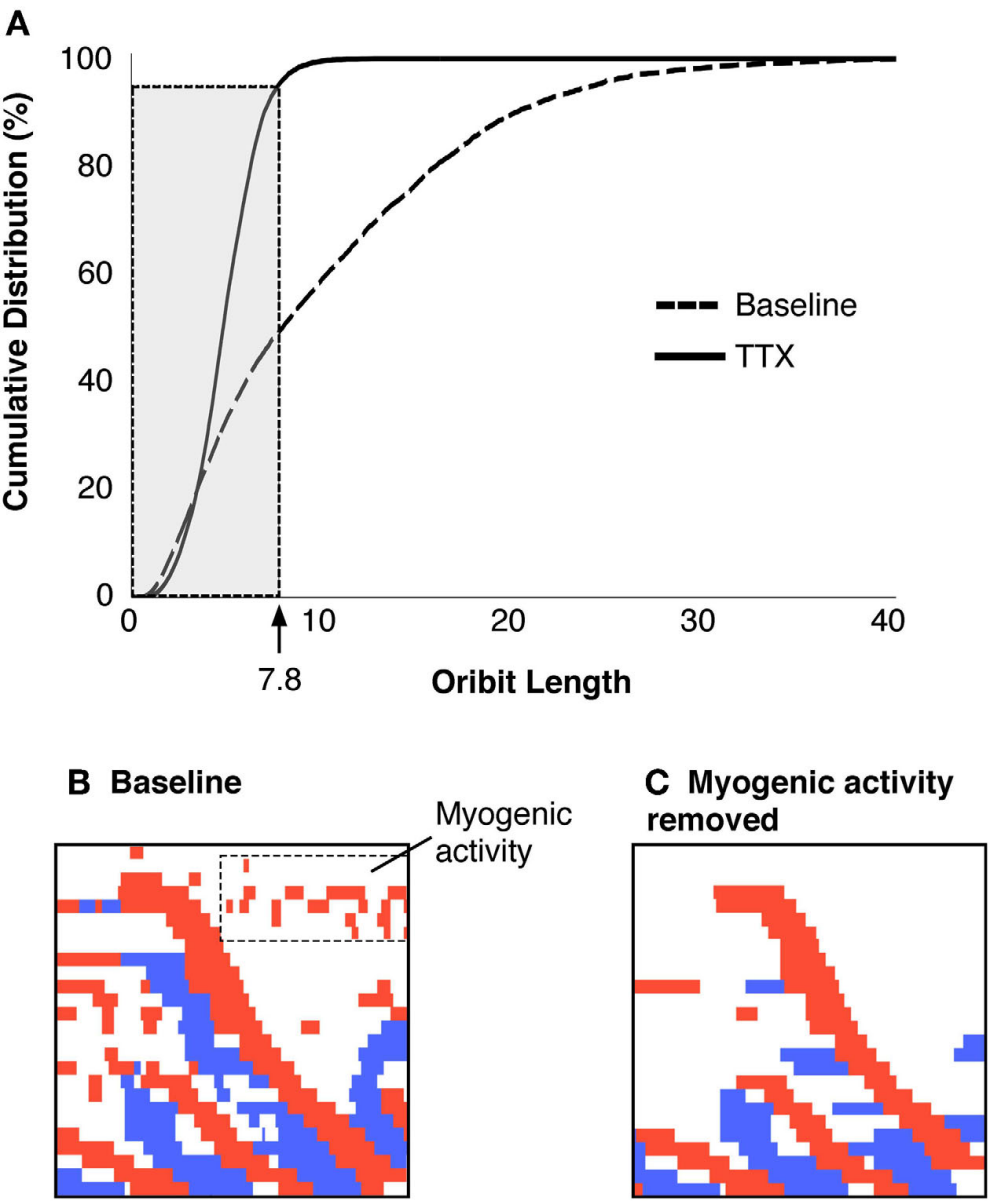
**(A)** Criteria to identify and remove myogenic activity. The cumulative distribution of orbital lengths calculated from preparations exposed to TTX are plotted (solid black line), with the cumulative distribution without TTX (dashed line, **A**). The cutoff that removed 95% of the myogenic activity was identified (value = 7.8). All points on the active-state map with orbit plots of 7.8 or less were removed from the data and remaining activity was considered to be neurogenic. The region shown in Figure 3F is shown with all activity displayed (**B**) and after myogenic activity is removed (**C**).

The mean diameter of the colon varied amongst the preparations, so the dimensions of the orbit plots were normalized, by dividing diameters and pressures in each preparation by the constants defining quiescence (see Figure 4 in Box 1; Wiklendt et al., 2013). The distributions of all orbit lengths (of pressure and diameter) were then plotted as cumulative frequency plots and the lengths/diameters that accounted for 95% of these data was arbitrarily defined as the threshold for myogenic activity (value of 7.8 in Figure 6A).

All orbits less than the threshold value (7.8 mm) were considered to be myogenic (Figure 6B) and were removed from active state analysis. These myogenic periods became part of the white regions in the active state maps (Figure 6C). Therefore, all remaining values could then be considered as reflecting active neurogenic states (excitation in red, inhibition in blue).

It is important to note that white areas on active-state-maps signify a lack of identifiable active states, but do not necessarily exclude neural activity occurring. Rather it indicates that if there is ongoing neural activity it is not the major determinant of the mechanical state of the muscle, at that site and at that moment. On the other hand, if there is a red or blue state (Figure 6) the likelihood that there is neural activity underlying those states is very high. It is also important to note that this methodology was developed specifically for the rabbit distal colon. Whether such a myogenic threshold value would be applicable in other species or other regions of the gut has not been established.

**Figure 7:**
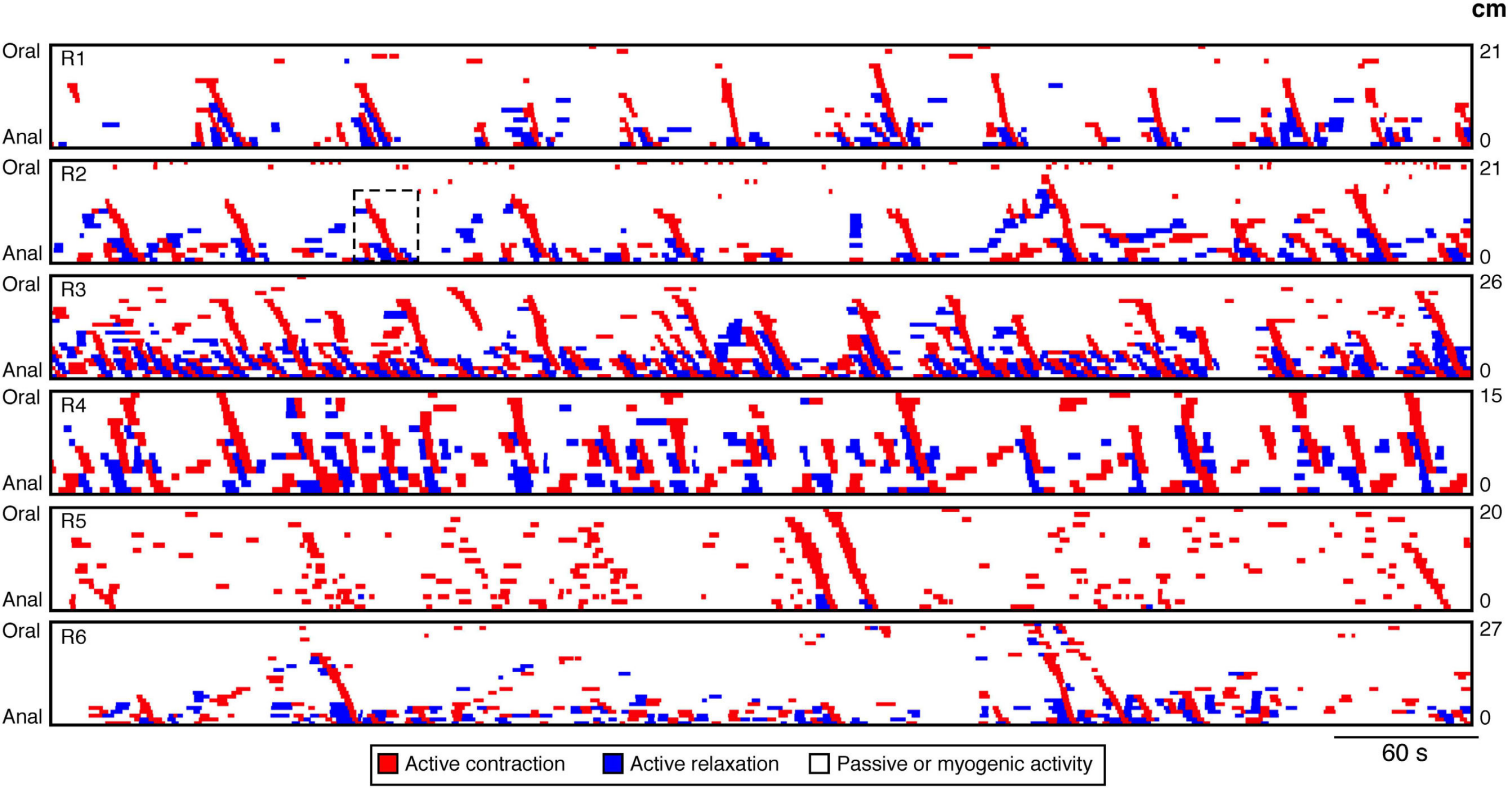
**DPMaps of neurogenic activity in the 6 rabbits**. The active contractions are shown in red and the active relaxations are shown in blue. The white represents the passive states and myogenic active states. From these maps all active propagating and non-propagating contractions were identified. The black hatched box on R2 indicates the region from which Figures 8, 11 were made.

The maps revealed an average of 25.8 ± 18.2 actively propagating contractions in 10 min per preparation and occurring at variable intervals of 30.19 ± 23.3 s. They traversed an average distance of 7.0 ± 1.2 cm, at a speed of 6.4 ± 0.8 mm/s. The average duration of an active contraction at any single point along the colon was 3.8 ± 1.7 s.

#### Initiation of neural peristalsis

From maps of active muscle excitation/inhibition (Figure 7), it is clear that not all active contractions propagated. This raises the question as to whether there is a difference in the amount of distension preceding the active propagating contractions in comparison to the active non-propagating contractions.

**Figure 8:**
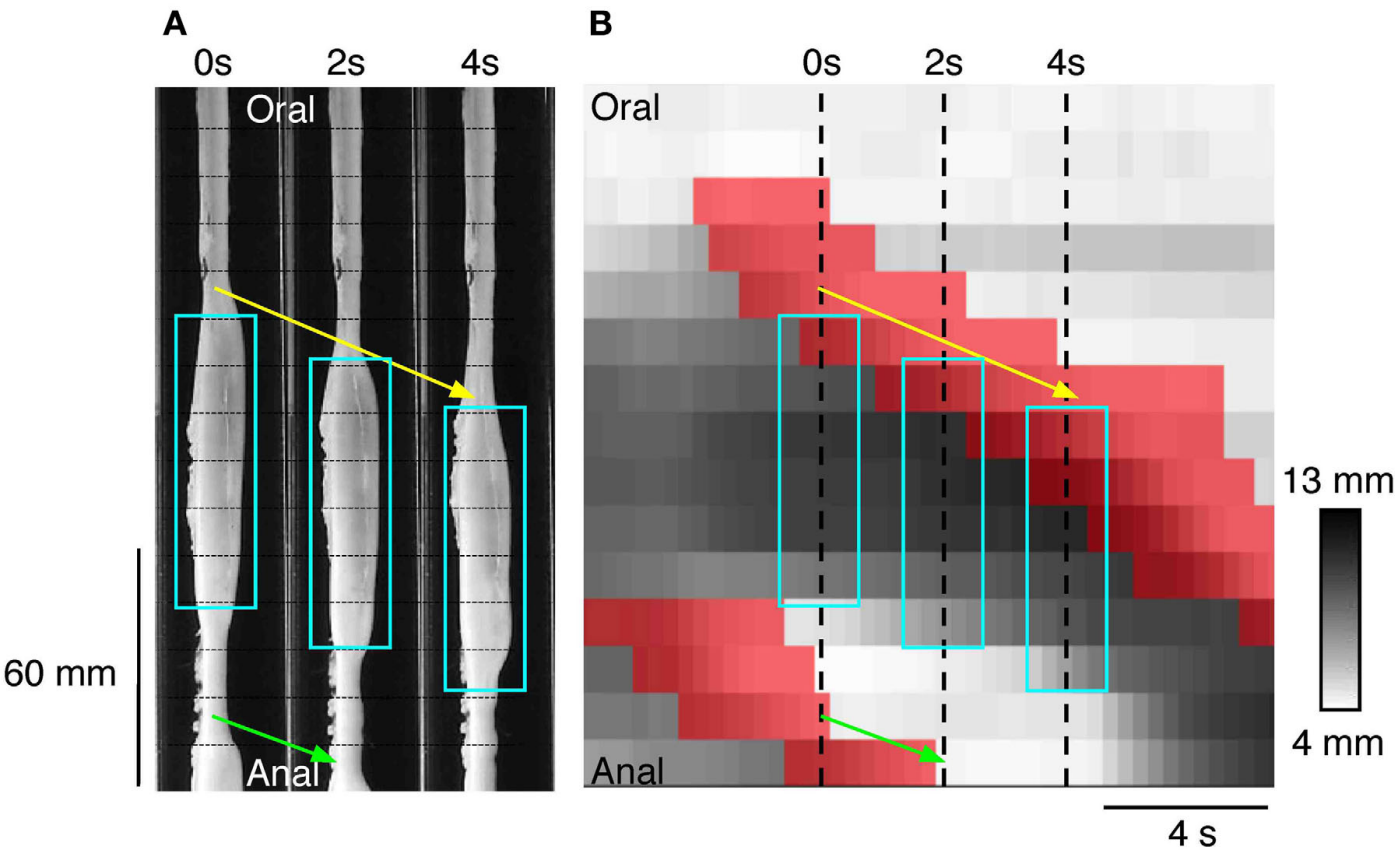
**(A)** Three silhouettes of the rabbit distal colon captured at 2 s intervals. The horizontal thin hatched lines on the silhouettes are spaced at 1 cm intervals and the fiber-optic pressure sensors are located between each of these lines. A propagating contraction can be seen on the silhouettes (connected by the yellow arrow) and this moves in an anal direction from the first to the third silhouette. Aqua rectangles surround the regions of distension. Part of a second propagating contraction (connected by the green arrow) can be seen at the bottom of panels 0 and 2 s. **(B)** The corresponding spatiotemporal DMap with the regions of active contraction shown in red. The three hatched vertical line represent the location of the three silhouettes shown in **(A)**. The areas in red a preceded by darkened regions (distended) on the DMap corresponding to the dilated areas within the white boxes in **(A)**. While it may appear as though there is a propagating contraction at both the oral and anal end of the aqua boxes in **(A)** it is important to note that at the anal end of the box no contraction has occurred. The red region at the bottom of the panel in **(B)** is actually associated with the second propagating contraction **(**green arrow in **A)**. At the anal end of each aqua box superimposed upon **(B)** there is no red region. This indicates that the gut is maintaining resting tone in its unstimulated state. Thus, by combining changes in diameter with changes in pressure we are able to determine when the gut is contracting and when the gut is at rest. This information cannot be determined from the DMap alone.

**Figure 9:**
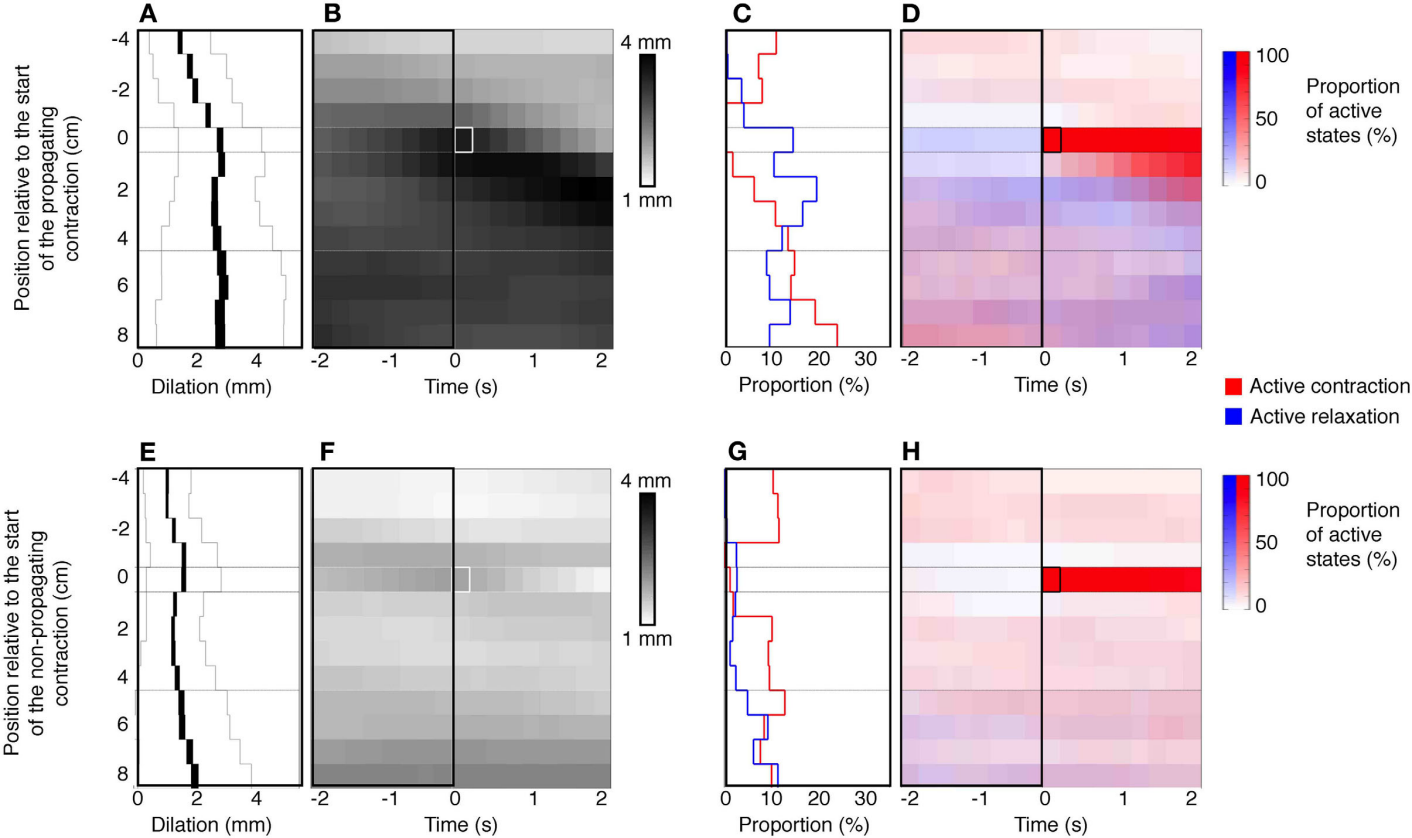
**Defining a stimulus for propagating contractions**. The top panels **(A–D)** represent the data from propagating contractions. The bottom panels **(E–H)** are collated from non-propagating contractions. The panels **(B,F)** represent the mean dilations (increase from baseline diameter) in a 4 cm region above and 8 cm region below the site of initiation of the active propagating **(**white box in **B)** and active non-propagating **(**white box in **F)** contractions. All active propagating and non-propagating contractions that originated within the proximal 4 cm or distal 8 cm of the preparation were excluded from this analysis. These panels also capture a 2 s time epoch leading up to and following the active contractions. **(A,E)** Indicated the mean dilation in the 2 s period prior to the initiation of the active contractions. The black line in **(A,E)** indicates the mean dilation at each point in time along the 12 cm region of colon and the thickness of the line indicates the 95% confidence interval. The thin gray lines indicated the standard deviation. The mean dilation immediately prior to the active propagating contractions is 2.7 mm which is significantly greater (*P* < 0.0001) than mean dilation prior to the active non-propagating contractions (1.6 mm). The right hand side of figure shows the relation that exists between the active states (contraction red and relaxation blue) and the active propagating **(C,D)** and non-propagating **(G,H)** contractions. Note that there is a significantly (*P* < 0.005) degree of active relaxation associated with active propagating contractions in comparison to active non-propagating contractions **(C,G)**.

An example of a propagating active contraction, taken from Rabbit 2 in Figure 7 (dashed line inset) is shown by the yellow arrow in Figure 8A. In Figure 8B, the red areas of active contraction have been superimposed onto a gray scale spatiotemporal map of diameters. From this combined map it is clear that at each zone of active excitation (red areas, Figure 8B) is temporally preceded by a distended region (dark gray region to its left). We therefore examined in all preparations the state of the muscle around the point of initiation. This was done by marking the exact site and moment of initiation of active contraction on spatiotemporal maps and then examining a box corresponding to muscle 4 cm above and 8 cm below the site of initiation and 2 s either side of the moment of initiation (Figure 9). Values in this box were averaged for all propagating active contractions, and compared to the average of all active contractions that failed to propagate (seen as isolated red spots, in Figure 7). In Figure 9, the point of initiation of propagating and non-propagating active contractions is shown by the small white or black boxes in all four spatio-temporal maps (Figures 9B,D,F,H). The region preceding active propagating contractions is substantially distended prior to the contraction (i.e., dark gray diameter in Figure 9B, to the left of the point of initiation). By comparison, active non-propagating contractions were preceded by much smaller distension (to the left of highlighted pixel in Figure 9F).

Quantitative analysis in Figures 9A,E shows that the amount of distension at the site of initiation of active contractions was significantly higher for propagating than non-propagating contractions (2.7 ± 1.4 mm vs. 1.6 ± 1.2 mm; *P* < 0.0001; Mann–Whitney).

There is one other feature of interest about the initiation of active propagating and non-propagating contractions. It is also clear that the average level of active inhibition anal to the point of initiation is greater for active propagating compared to non-propagating contractions (compare blue density in the area below red pixels in Figures 9D,H). Quantitative analysis shows that active inhibition of the muscle at the site of initiation in the seconds preceding the abrupt onset of contraction (Figures 9C,G, left of highlighted pixel) is much greater for active contractions that propagate compared to those that do not (*P* < 0.005; Fisher's Exact Test).

#### Propagation of peristaltic contractions in discrete boluses

In Figure 8A it is noticeable that the darker (dilated) regions preceding the red areas (neurally dependent active contractions) is due to the formation of a liquid bolus, as shown in the silhouettes (white boxes), with visible contractions of the circular muscle just oral to the bolus.

Formation of discrete bolus during active propagating contractions (neural peristalsis) in the rabbit colon was common and they traveled over several centimeters along the preparation. According to the neuromechanical hypothesis, originally formulated in a simple form by Bayliss and Starling (Bayliss and Starling, 1899) the bolus should be associated with active contraction at its oral end and active relaxation at its aboral end.

To test this we compared states of the muscle associated with propelled and stationary boluses using combined spatio-temporal maps.

Each bolus was identified with automated analysis (Matlab), defined by a dilation of the gut greater than 2 mm, above minimum occluded diameter that persisted over a time window of at least 0.5 s. The length of the bolus was calculated from the points at the oral and anal edges, which exceeded 2 mm above minimal diameter. Bolus propulsion velocity was calculated by smoothed interpolation of the locations of the oral and anal edges over time. As the manometric pressure sensors were spaced at 1 cm intervals, intraluminal pressure was correlated with the averaged colonic diameter in 1 cm bins along the length of the bolus.

When the diameter at a fixed reference location is measured as a bolus passes, the diameter gradually increases as the anal edge of the bolus arrives, then diameter would remain roughly constant, then gradually decrease as the oral edge passes. This has to be taken into account in measuring contractions since a rapidly moving bolus will show sharp increase and then decrease in diameter. Conversely a slowly moving bolus is associated with slow changes in diameter, measured at any fixed point in the preparation. If the bolus moves too slowly it begins to fall into a period of quiescence, as defined by our current model (see Box 1).

**Figure 10:**
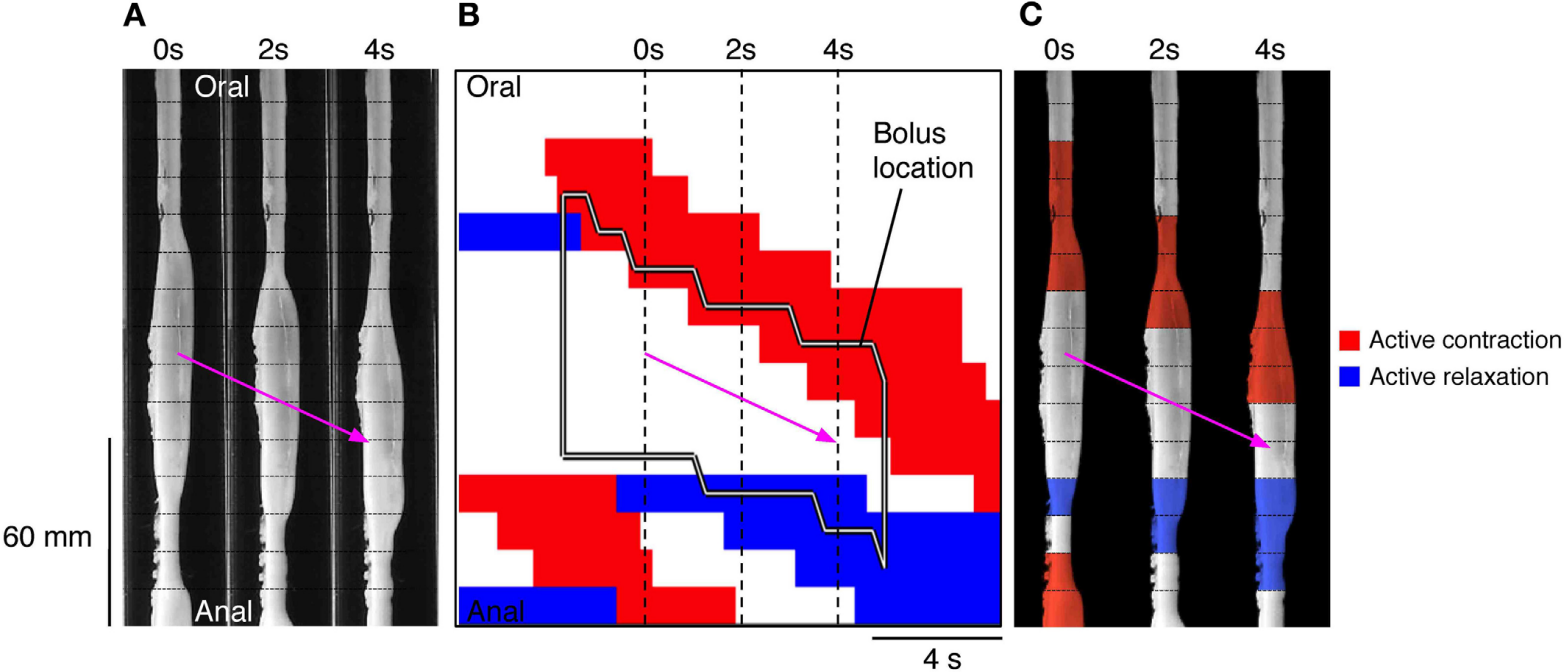
**(A)** Using the same panel as shown in Figure 8 we have now added an outline of the bolus **(**black line shape in **B)** and the active states of neurogenic activity associated with the bolus movement. In **(B)** the red indicates active contraction at the oral end of the bolus, and blue indicates an active relaxation at the anal end of the bolus. The vertical hatch lines in **(B)** indicate the location of the silhouettes shown in **(A)**. These active states are then superimposed upon these three silhouettes **(C)**. The magenta arrow indicates the direction of the bolus movement. As can be seen in **(C)** the bolus movement is associated with active contraction (red) at the oral end of the bolus and active relaxation (blue) at the anal end.

**Figure 11:**
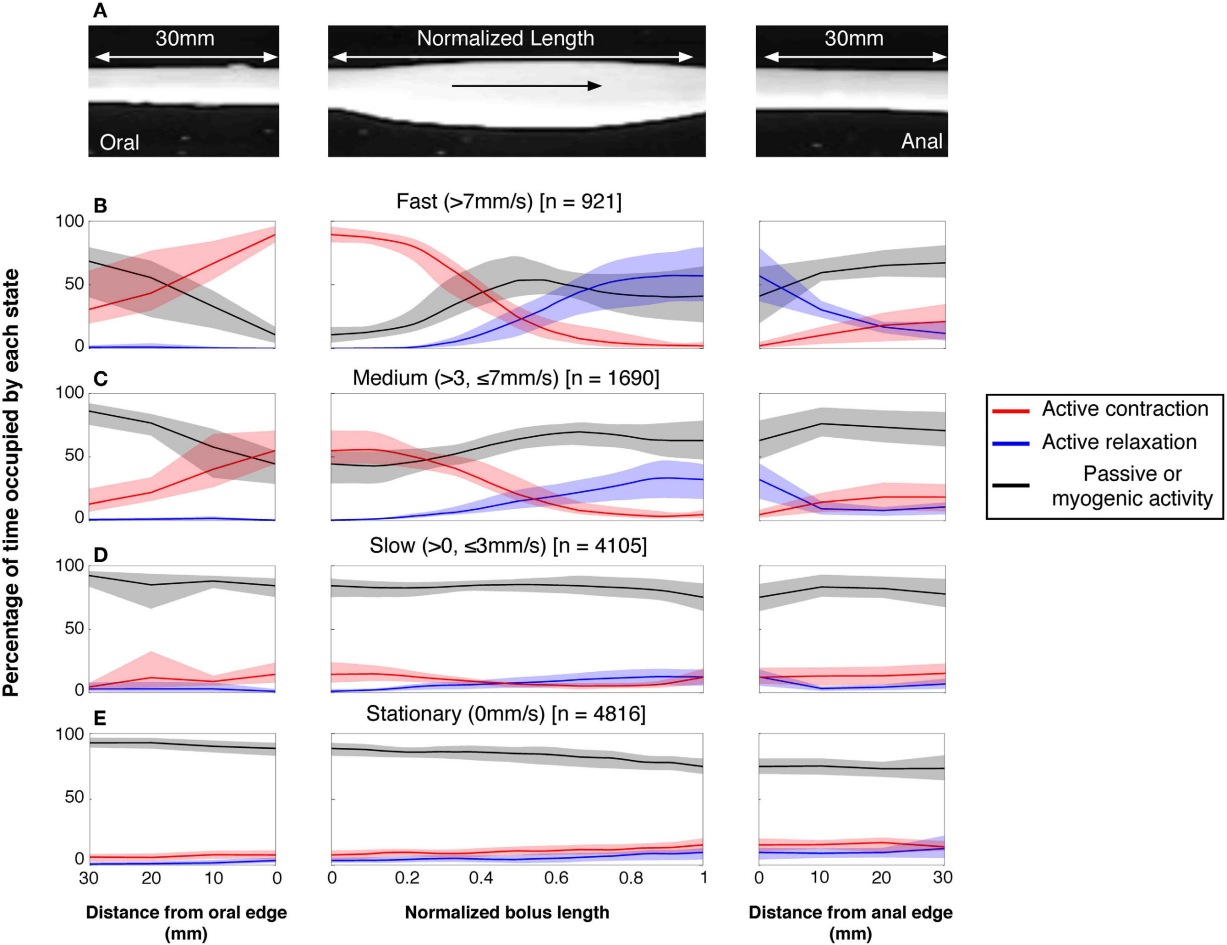
**The neurogenic active states associated with all moving and stationary boluses in the rabbit distal colon. (A)** The active states were calculated on a frame by frame basis in a 30 mm section of gut before and after the bolus and throughout the actual location of the bolus. In **(B–E)**, the red lines indicate the percentage time occupied by active contractions at each frame of analysis. The blue lines indicate the active relaxation and the black lines represent all other muscular states (passive and myogenic). The color-coded shading around each line represents the 95% confidence interval for the mean percentages over each rabbit. The speed of propagation has been divided into 3 bins **(B)** > 7 mm/s; **(C)** > 3 ≤ 7 mm/s; **(D)** > 0 ≤ 3 mm/s with stationary boluses shown in **(E)**. The number in the square brackets indicate the number of analyzed frames in each velocity bin. As can be seen at the fastest velocity there is a strong association between active contractions at the oral end and active relaxation at the anal end. As the speed of movement slows, this association drops and at the slowest speeds the active contractions and relaxations are almost gone. This loss of association is due, at least in part, to current analytical model. As the bolus movement slows our model begins to define the bolus as being stationary (see Figure 4). The stationary boluses have no association with active contractions or relaxations.

Once the moving boluses were identified we used orbital plots at each point to determine whether their aboral propulsion was associated with active contraction and/or active relaxation. In Figure 10B this relationship is shown for the aboral propulsion of a single bolus. This example is fairly typical: active contraction (red) occurred at the oral end of the bolus (and extended further orally) and active relaxation (blue) occurred at the anal end, also extending beyond it. By normalizing the length of the bolus, it was possible to calculate the mean distributions of excitation and inhibition along boluses, which were grouped according to their speed of movement (see Figure 11).

The vast majority of rapidly moving boluses (>7 mm/s) were associated with propagating active contractions at their oral side (93.6%; the apparent absence of active contraction is the result of the algorithm chosen to identify active contractions). In these boluses neural active relaxation at the anal end occurred in 57% of the times (Figure 11B middle panel). In the middle part of each bolus there was a mixture of passive and active mechanical states. The active contractions started up to 30 mm oral to the bolus. (Figure 11B left panel) and the active relaxation extended up to 30 mm ahead of the advancing bolus (Figure 11B right panel). In slower-moving boluses (>3, <7 mm/s) the clear separation of excitation and inhibition diminished (Figure 11C). At the slowest speeds (<3 mm/s) no significant difference in active muscle states occurred along the bolus (Figure 11D). However, this may be the result of the algorithm in our model used to identify periods of quiescence. At these slowest velocities our current model begins to identify the bolus as being stationary. As can be seen, stationary boluses were not associated with localized excitation or inhibition (Figure 11E). Measurements at random points in the spatiotemporal maps (which mostly missed actual boluses) confirmed that the corresponding distribution of active excitation and inhibition is random and thus indicates that the polarized distribution of active states on the boluses is not due to systematic errors.

The question arises as to why not all boluses formed and propelled by the peristaltic contractions (red steaks in Figure 7) are associated to active relaxation at their anal ends (blue areas in Figure 7). Even at the fastest bolus speed 43% were not associated with active relaxation (Figure 11B middle panel). Distension has been shown to be an effective stimulus to initiate peristaltic contractions in colon of most species (Auer and Krueger, 1947; Hukuhara and Miyake, 1959; Hukuhara and Neya, 1968; Costa and Furness, 1976; Jule, 1980; Grider and Makhlouf, 1986; Messori et al., 1995; Bjornsson et al., 1998; Bian et al., 2003; Spencer et al., 2003, 2011). We hypothesized that the degree of distension may play an important role in the activation of the descending inhibitory reflex pathways.

**Figure 12:**
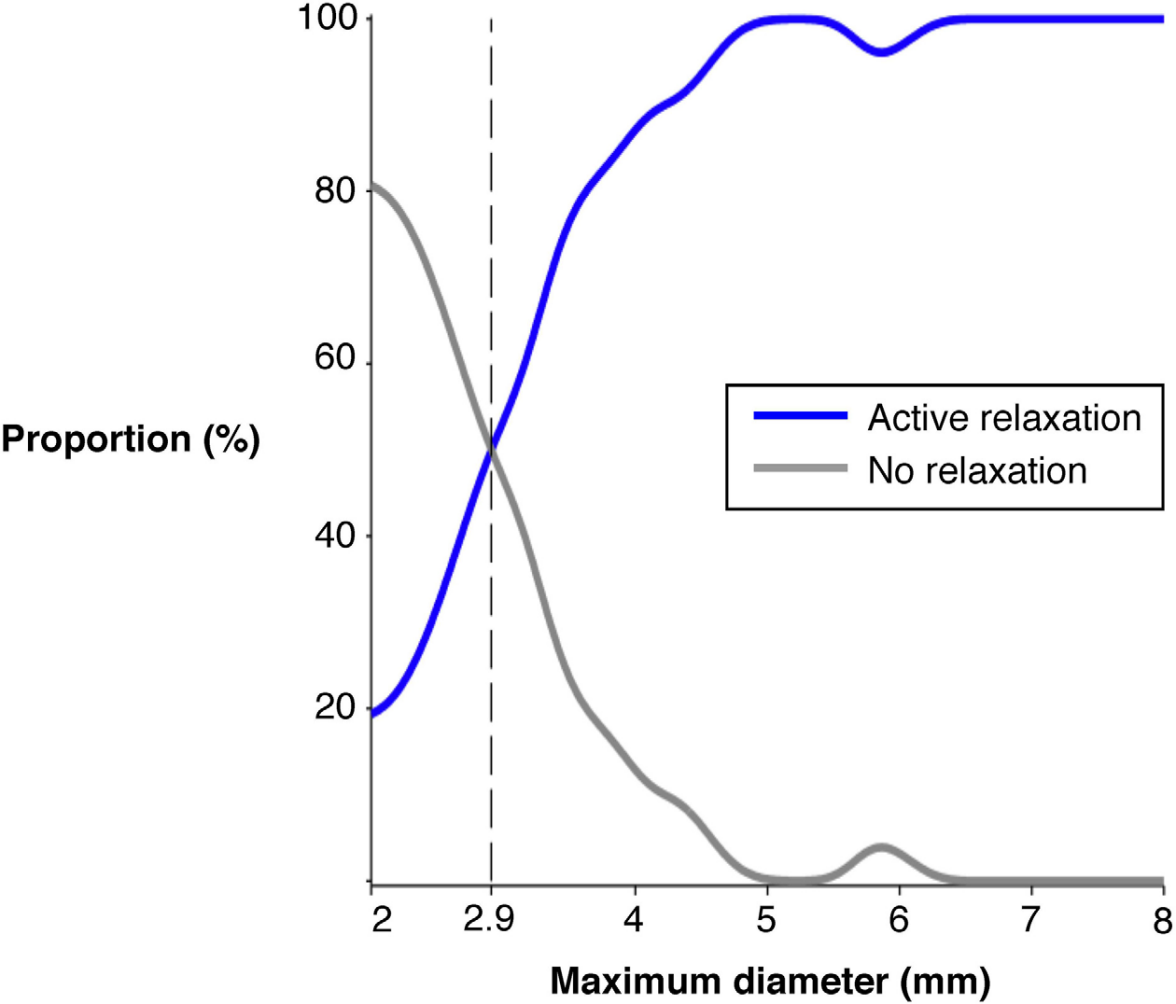
**The proportion of fast (>7 mm/s) moving boluses associated with active inhibition at the anal end of the bolus**. The maximal diameter size refers to the increase above the minimum occluded diameter and this was calculated at each frame during the movement of the bolus. As the diameter increases the proportion of boluses associated with active relaxation increase. Once the diameter exceeds 5 mm above the minimum occluded diameter nearly all boluses are associated with active relaxation.

We therefore examined the diameter of the fast moving boluses propelled by active contractions. In Figure 12 it can be seen that there is an excellent relation between maximal distension diameter and occurrence of active relaxation at the anal side of the bolus. While only 20% of the boluses of 2 cm diameter elicited anal relaxation, half of the boluses of 2.9 cm diameter did and practically all boluses greater than 5 cm did. We also examined the length of the bolus to determine if that played a role. However, there was no difference in the proportion of active relaxations ahead of advancing boluses of 20 mm or less length (71.6%) and that of boluses more than 20 mm long (81.0 %) (standard two by two table; chi-squared *P* = 0.1166).

## Discussion

According to the neuromechanical loop hypothesis, first proposed in a simplified manner by Bayliss and Starling (Bayliss and Starling, 1899), peristaltic contractions are initiated by a bolus that distends the intestinal lumen. This distension activates polarized enteric neural pathways, with excitation local and oral to the site of distension, and inhibition anally. The localized oral excitation propels the bolus, assisted by the downstream inhibition, leading to a new set of polarized reflexes being activated that repeat the process. In this way a wave of circular muscle contraction propagates along the colon, pushing content ahead of it.

Although we have used a method that does not directly record the activity of enteric motor neurons, our method does identify areas of smooth muscle that are being actively excited or inhibited—which is effectively attributable to input from enteric motor neurons activated by polarized reflex pathways. This provides evidence to support the “neuromechanical loop hypothesis” for intestinal peristalsis.

### Initiation of peristaltic contractions

The actual mechanisms by which distension initiates peristaltic contractions are not fully understood. In our closed preparations, passive distension resulting from the redistribution of fluid after each peristaltic contraction is a likely cause. Our results show that active propagating contractions are preceded by localized distension greater than that preceding active non-propagating contractions (see Figures 9 A,E). This confirms the observations that distension is an effective stimulus to initiate peristaltic contractions in colon of most species (Auer and Krueger, 1947; Hukuhara and Miyake, 1959; Hukuhara and Neya, 1968; Costa and Furness, 1976; Jule, 1980; Grider and Makhlouf, 1986; Messori et al., 1995; Bjornsson et al., 1998; Bian et al., 2003; Spencer et al., 2003, 2011).

It is also not clear why peristaltic contractions often initiate at seemingly unremarkable points along the intestine (i.e., not always at the oral end of the specimen). In addition to passive distension, the sites of initiation can be actively inhibited prior to the initiation of contraction. This neurally-mediated inhibition at the moment and site of initiation obviously cannot be due to descending inhibition triggered by an advancing bolus. More likely this represents activation of the localized accommodatory reflex pathways triggered by fluid distension, as reported previously in several preparations of gut (Cannon and Lieb, 1911; Paton and Vane, 1963; Furness and Costa, 1977; Desai et al., 1991; Waterman et al., 1994a; Hennig et al., 1997; Bogeski et al., 2003). However, our data clearly indicated that active relaxation at the site of initiation is not always required (see Figures 9C,D). It is likely that the areas of initiation of peristaltic contractions represent areas of maximal distension by both passive and active dilation, again consistent with the hypothesis that distension is a major physiological trigger for neural peristalsis.

### Propagation of peristaltic contractions

Our results support the neuromechanical loop hypothesis that propulsion of boluses is due to ongoing activation of polarized enteric reflex pathways by the advancing bolus. The majority of traveling boluses were associated with an area of active contraction at their oral end and above it. The neurally driven active contraction must result from the excitatory motor neurons to the circular muscle by the ascending excitatory reflex pathways activated by the distending bolus.

The neuromechanical hypothesis also predicts that the ongoing distension by the propelled bolus activated descending inhibitory reflex pathways resulting in anal relaxation. Indeed our results show that the occurrence of neurally driven anal relaxation was graded by the diameter of the bolus. Practically all larger diameter boluses (traveling at >7 mm/s) were associated with an area of active inhibition of the muscle at their aboral end and further aborally, while some boluses of smaller distensions did not produce anal relaxation (Figure 12).

Thus while an active propagating contraction oral to the bolus is an essential component of peristalsis, the anal relaxation accompanying it may play a permissive role only. If the bolus diameter is small enough, propulsion can occur with presumably a lack of downstream resistance. As the bolus diameter increases the force required to move the bolus would increase. The larger boluses are likely to activate more effectively descending inhibitory neurons resulting in active relaxation at the anal end of the bolus, thereby allowing the bolus to travel down the colon. Somewhat surprisingly the length of the bolus does not appear to contribute to the incidence of anal relaxations.

These results suggest some significant differences between the dynamics of activation of the ascending excitatory reflex pathways and descending inhibitory pathways during peristalsis. The active contractions appear to be initiated by mechanical dilation at a threshold level in an all-or-nothing manner while the anal active relaxations are graded.

In the middle of the advancing bolus there was no indication of activation of enteric reflex pathways and much of the mechanical states appeared to be passive. Thus, the peristaltic contractions displace fluid from the already-contracted region, causing distension (bolus), which activate the polarized enteric reflex pathways. The process is repeated sequentially resulting in the bolus propulsion by the peristaltic contraction as predicted by the neuromechanical loop hypothesis. Comparison with measurements of muscle state at random points confirmed that the characteristic distribution of excitation and inhibition around boluses did not occur by chance.

As mentioned in the introduction, the existence of polarized reflex pathways along the intestine has been well-established (Auer and Krueger, 1947; Hukuhara and Miyake, 1959; Hukuhara and Neya, 1968; Costa and Furness, 1976; Jule, 1980; Grider and Makhlouf, 1986; Messori et al., 1995; Bjornsson et al., 1998; Bian et al., 2003; Spencer et al., 2003, 2011). Extensive anatomical studies, in a few model preparations, have confirmed that enteric neuron show distinctive polarization too (Costa et al., 1996) and modeling has confirmed that these can account for ascending excitatory reflexes (Thomas et al., 1999). One detail is important: polarized pathways are apparent even at the level of circular muscle motor neurons. Inhibitory motor neurons typically project axons a short distance anally before innervating the muscle. However, excitatory motor neurons project both directly to circular muscle overlying their cell bodies, as well as to more oral regions. This has been shown in guinea pig small intestine and stomach as well as in the human colon (Brookes et al., 1991, 1998; Porter et al., 1997) Putative mechanosensory neurons have predominantly circumferential projections (Bornstein et al., 1991). Put together with the projections of motor neurons, this may well-explain why circular muscle both within the oral end of a distended bolus, is actively excited by enteric circuits (see Figures 10, 11). Both excitation and inhibition extend well-beyond the boundaries of the bolus: this is likely to be due to activation of polarized interneuronal pathways which have longer projections than motor neurons (Costa et al., 1996; Brookes, 2001a,b).

### Termination of peristaltic contractions

The neuromechanical hypothesis also predict that if the loop is interrupted at any stage, peristalsis should cease. One factor is the duration of activation of the enteric reflex responses. The duration of the active contraction at any point along the colon was approximately 4 s. This limited duration was often observed despite the continuing presence of an ongoing distending stimulus. This transient nature of the activation of ascending excitatory reflexes has been demonstrated in other preparations (Costa and Furness, 1976; Tonini et al., 1996) and possibly reflects an intrinsic dynamic property of enteric neural circuitry. The transient nature of polarized excitatory reflexes would lead to an expectation that a stationary bolus would not be associated with polarized activation of excitatory and inhibitory enteric reflex pathways. Our analysis shows that this is the case (Figure 11E). The transient nature of the active contraction results in the fluid to flow passively back after the peristaltic contractions, thus reducing the distension stimulus. Consistent with the neuromechanical loop hypothesis, unless there is a continuing supra threshold mechanical distension by the advancing bolus, the transient activation of the enteric excitatory neural circuits ceases to sustain peristaltic contractions.

### Other mechanisms may operate during peristaltic contractions

The evidence presented here that propulsive peristaltic contractions are due to the polarized activation of excitatory and inhibitory enteric neural pathways by mechanical stimuli does not exclude the possibility that other mechanisms also contribute.

The circular muscle contraction initiated by excitatory motor neurons may activate tension-sensitive enteric primary afferent neurons (Kunze and Furness, 1999). These neurons could then provide self-sustaining excitation of the circular muscle aborally by activating descending excitatory enteric pathways (Spencer et al., 1999).

In the guinea-pig small intestine a similar mechanism was proposed for peristalsis evoked by fluid distension, with the imbalance between excitation and inhibition at the oral end determining the site of origin (due to the polarity of excitatory and inhibitory pathways), and subsequent propagating of peristaltic contractions (Waterman et al., 1994b).

### Additional mechanisms of initiation of peristaltic contractions

Another feature that requires comment is the nature of the mechanisms behind the intermittent occurrence of the peristaltic contractions during maintained distension (Figure 7). At the end of each peristaltic contraction, redistribution of fluid content occurs. Yet this redistribution of fluid to the proximal end of the preparation does not immediately trigger another peristaltic contraction. This intermittent nature of the initiation of peristaltic contractions may be due to cyclic changes in the threshold for initiation of peristaltic contractions. Cyclic, neurally-mediated motor activity has been observed in the colon in many species (Christensen et al., 1974; Costa and Furness, 1976; Sarna, 1986; Spencer et al., 1998b; Spencer, 2001; Smith et al., 2003). The mechanisms underlying the cyclic motor activity, which usually travels slowly aborally (thus often named “colonic migrating motor complexes”), are still under investigation (Spencer et al., 1998a; Mule et al., 1999; Gonzalez and Sarna, 2001).

The intermittency of peristaltic activity observed in our study and in many other intestinal preparations (Costa et al., 2013a), could be due to low frequency cyclic changes in neural circuit excitability, a myogenic controlled system or both. Excitation may involve slow cyclic changes in the network of ICCs at the myenteric plexus level, and that these provide a “myogenic” background on to which superimposed cyclic neural activity work synergistically to produce the full propagating and propulsive motor pattern. Such a hypothesis of myogenic initiated peristalsis has recently been described in the rat colon (Huizinga et al., 2011; Chen et al., 2013). Our current data cannot exclude or support such a hypothesis and further investigations will be required to clarify the cellular mechanisms of such intermittency.

Finally the rabbit distal colon normally contains formed feces. Bayliss and Starling in their 1900 investigation of colonic peristalsis in the dog and rabbit (Bayliss and Starling, 1900), suggest that polarized reflex pathways are responsible for the propulsion of a balloon (solid bolus). It likely that similar pathways and mechanisms are involved is propulsion of solid boluses. However, at present we are unable to run a solid bolus without interfering with the fiber-optic catheter. We are confident that with suitable technical modifications the relation between muscle states and the movement of a solid bolus may form the basis of profitable future work.

## Author contributions

Phil G. Dinning, Lukasz Wiklendt, and Marcello Costa; study concept and design, analysis and interpretation of data, draft and critical review of manuscript. Simon J. H. Brookes, Taher Omari, John W. Arkwright, and Nick J. Spencer critical manuscript review and interpretation of data.

### Conflict of interest statement

The authors declare that the research was conducted in the absence of any commercial or financial relationships that could be construed as a potential conflict of interest.
